# Seroprevalence of Yellow fever, Chikungunya, and Zika virus at a community level in the Gambella Region, South West Ethiopia

**DOI:** 10.1371/journal.pone.0253953

**Published:** 2021-07-08

**Authors:** Getahun Asebe, Daniela Michlmayr, Gezahegne Mamo, Woldaregay Erku Abegaz, Adugna Endale, Girmay Medhin, James W. Larrick, Mengistu Legesse

**Affiliations:** 1 Department of Veterinary Microbiology, Immunology and Public Health, College of Veterinary Medicine and Agriculture, Addis Ababa University, Bishoftu, Ethiopia; 2 College of Agriculture and Natural Resources, Gambella University, Gambella, Ethiopia; 3 Division of Infectious Diseases and Vaccinology, School of Public Health, University of California, Berkeley, Berkeley, California, United States of America; 4 Department of Microbiology, Immunology & Parasitology, School of Medicine, College of Health Sciences, Addis Ababa University, Addis Ababa, Ethiopia; 5 Aklilu Lemma Institute of Pathobiology, Addis Ababa University, Addis Ababa, Ethiopia; 6 School of Medicine, College of Medicine and Health Sciences, Dire Dawa University, Dire Dawa, Ethiopia; 7 Panorama Research Institute, Sunnyvale, California, United States of America; CEA, FRANCE

## Abstract

Yellow fever (YF), Chikungunya (CHIK), and Zika(ZIK) are among re-emerging arboviral diseases of major public health concern. Despite the proximity of the Gambella Region to South Sudan where arboviral cases have been recorded repeatedly the current epidemiological situation is unclear in this part of southwest Ethiopia. Therefore, we conducted a community-based seroprevalence survey of YF virus (YFV), CHIK virus (CHIKV), and ZIK virus (ZIKV) infections in two selected districts. A cross-sectional study was conducted in two locations of the Gambella region (Lare and Itang) to investigate the seroprevalence of these viruses’ infections. Blood samples were collected from the study participants and screened for IgG antibodies specific to YFV and CHIKV infections using enzyme-linked immunosorbent assays (ELISA). For the detection of ZIKV specific IgG antibodies, Blockade-of-binding ELISA was used. Data were analyzed using the STATA version 13.1 Softwares. A total of 150 individuals (96 males and 54 females, age ranging from 18 to 65 years, mean age ± SD = 35.92 ± 10.99) participated and provided blood samples. Among the 150 samples 135, 90, and 150 were screened for YFV, CHIKV, and ZIKV, respectively. Hence, 2.9% (95% CI: 1.1–7.7%), 15.6% (95% CI: 9.3–24.8%), and 27.3% (95% CI: 20.7–35.3%) of samples tested positive for IgG antibodies to YFV, CHIKV, and ZIKV infections, respectively. Among the individual seropositive for ZIKV, YFV and CHIKV, only six, one and three had a history of residence outside the Gambella region respectively. Agro-pastoral occupation was significantly associated with a higher prevalence of IgG against CHIKV (AOR = 14.17; 95%CI: 2.30, 87.30) and residency in the Lare district (AOR = 11; 95%CI: 3.31, 39.81) was found to be significantly associated with a higher prevalence of IgG against ZIKV. Our findings revealed the occurrence of YFV, CHIKV and ZIKV infections in the study locations.

## Introduction

Emerging and re-emerging mosquito-borne viruses such as Yellow fever virus (YFV), Chikungunya virus(CHIKV), Dengue virus, and Zika virus (ZIKV) have become major public health concerns in tropical and subtropical countries [1–3]. Climate change, vector adaptations, urbanization, migration of people, and expansion of agricultural activities to sylvatic areas are among the factors contributing to the spread of arboviruses to a wider range of geographical areas [4].

YFV is a re-emerging arbovirus that belongs to the genus *Flavivirus* [5]. YFV is transmitted by several mosquitoes species (spp) including *Aedes* spp., *Haemagogus* spp., and *Sabethes* spp [6]. Africa accounts for ~90% of the global YFV infection cases. YF is an endemic and intermittently epidemic disease affecting a wide range of the continent [7]. Recently, African countries like Nigeria [7], Uganda [8], Ghana, Chad, Guinea, the Republic of the Congo [8], and Angola [9] reported YF outbreaks. In Ethiopia, YF outbreaks have repeatedly occurred since the 1960’s resulting in over 30,000 deaths in the southern part of the country [10]. In 2013, a YF outbreak re-occurred in the South Omo Zone of southern Ethiopia resulting in many deaths [3]. Recently, another YF outbreak was reported from the Gurage and Wolayita areas of southern Ethiopia [11].

CHIKV of the genus *Alphavirus* is transmitted through the bite of an infected female mosquito belonging mainly to *Ae*. *aegypti* and *Ae*. *albopictus*. CHIK is a zoonotic disease widely distributed in many tropical and subtropical regions of sub-Saharan Africa [12, 13] including Sudan [14], Kenya [15], Tanzania [16], and Uganda [17]. In June 2016, Ethiopia confirmed its first documented case of CHIK from the Suuf kebele, Dollo Ado district of the Somalia regional state of Ethiopia bordering the Mandera region of Kenya, where a CHIKV outbreak was ongoing [18, 19]. Additionally, CHIK cases were reported from the Dire Dawa and the Afar regions, Eastern Ethiopia [20, 21]. Recently, a study by Endale and his co-authors reported a high seroprevalence (43.6%) of CHIKV infections in the South Omo region of southwest Ethiopia which adjoins the current study location (Gambella region, Southwest Ethiopia) [22]. There is no data about CHIKV circulation in the Gambella region.

ZIKV is a *Flavivirus* first isolated in 1947 from a rhesus monkey resident in the Zika forest of Uganda [23]. ZIKV infection of humans was first reported from Uganda and Tanzania in 1952 [24]. Like CHIKV, ZIKV transmission occurs via the bite of an infected female mosquito belonging mainly to *Ae*. *aegypti* and *Ae*. *Albopticus* [25]. Recent reports confirmed sexual transmission of ZIKV and transmission through blood transfusion [26], and also vertical transmission from mother to the fetus [27]. There is no data about ZIKV circulation in the Gambella region.

The Gambella Region has been selected as a study site because it is adjacent to the South Omo area in Ethiopia where YF is endemic [3]. This region is also proximal to South Sudan and Kenya where many arboviral cases are frequently reported [28–30]. Besides the geographical proximity of the area, the risk of introduction of disease is expected to increase due to the free movement of domesticated animals, wild game, and migration of people between these areas.

No epidemiologic information on arboviral diseases in the Gambella region is available, due to the lack of community- or health facility-based studies. We now report the first community-based seroprevalence study of YFV, CHIKV, and ZIKV infections in the Gambella region in the South West of Ethiopia.

## Materials and methods

### Study area and population

This study was conducted in the Gambella Region of South West Ethiopia between late October 2018 and mid-June 2019. Gambella is located at an elevation between 1,000 to 2,000 meters above sea level (masl) in the East, to 500–900 masl in the center, and 300–500 masl in the West [31] with a recent population projection estimated to be 435,999 [32]. Lare and Itang special districts were selected as study sites for this research. Lare is located at 300–500 masl in the West and borders South Sudan. Itang’s special district borders are Lare and South Sudan to the West. These districts were purposely selected because of their proximity to neighboring countries like South Sudan and Kenya where outbreaks of different arboviruses have been reported [33, 34]. Specific sub-districts were also considered during selection based upon their proximity to refugee camps, as well as the ecology of the area such as forest, water bodies, population density, and the occurrence of mosquito vectors and prevalence of arboviral infections in the bordering area.

**Fig 1:**
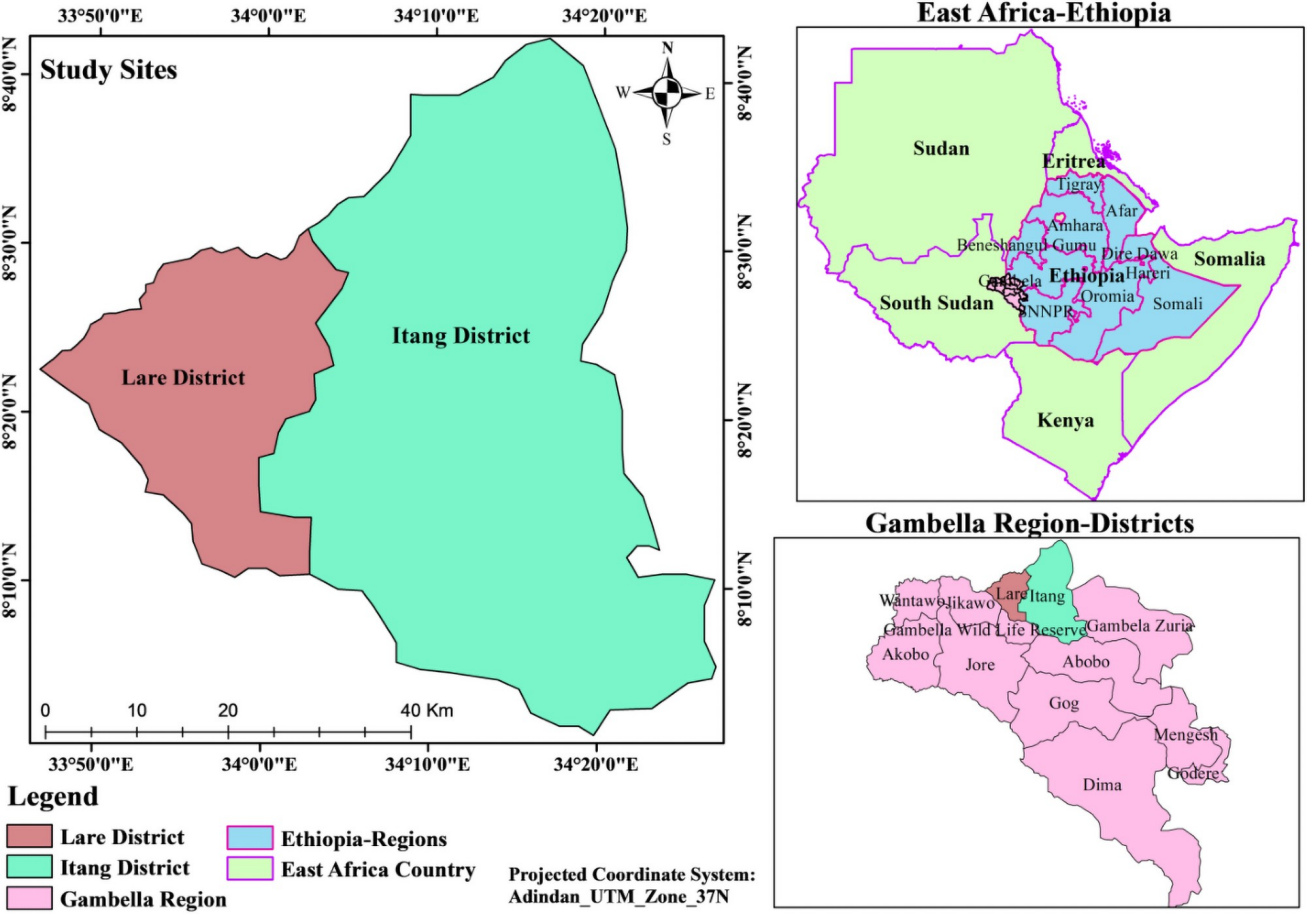
Map of the study site (Lare district and Itang special districts).

These districts host many refugees from South Sudan and migratory pastoralists from South and North Sudan (commonly called “Fallata” or “Fulani”). In addition, native pastoralists of the Lare district traditionally travel far from their villages to the adjacent territories of South Sudan for social activities and in search of pasture. In this study, six “kebeles” (the lowest administrative structure in a district in Ethiopia) from the Itang special district, which are closer to the refugee camps, and four “kebeles” from Lare district, contingent to the border of South Sudan were included (Fig 1).

### Study design, sample size, and sampling techniques

A community-based volunteer study was designed. Inclusion criteria were: age ≥18 years, ability to give written consent and respond to the questionnaire, known resident in the “kebele” for more than six months, apparently healthy (with no current illness, not suffering from obvious medical and psychiatric problems), and able to provide 3 ml blood sample. The locations of and proximity to the refugee camps, community movements with bordering territories, and the adjacent sub-districts were documented. Pregnant women, sick individuals, refugees, and visitors to the area were excluded from the study. Refugees were excluded because the antibodies detected in these vulnerable groups may have resulted from a previous infection in their country of origin and would certainly not measure exposure to the disease in the study area. During the sampling procedures, sub-districts were purposively selected seeking areas with a higher mosquito burden.

One hundred fifty (150) community members gave written consent and provided a 3 ml venous blood sample once during the study period.

All 150 blood samples were screened for IgG specific to ZIKV using the blockade-of-binding (BOB) technique. Blood samples from 135 for YFV and 90 participants were also screened for IgG specific to YFV and CHIKV, respectively, using indirect enzyme-linked immunosorbent assay (ELISA) techniques.

### Data collection and laboratory investigation

Data including socio-demographic characteristics, duration of stay in the study area, history of residence/travel in other countries, and history of vaccination against YFV or other arboviruses were collected using a structured questionnaire (S1 and S2 Questionnaires). Venous blood samples of each study participant were collected using serum separator vacutainer test tubes uniquely labelled for each person. To increase the amount of serum and maximum separation the test tubes were centrifuged for 15 minutes at 3400 rotations per minute, and the serum separated and stored at -20°c until screened for immunoglobulin G (IgG) antibodies against YFV and CHIKV using a sandwich ELISA assay (Abbexa Ltd, Cambridge UK) [35] as described in detail by the manufacturer. In brief, a 96 well plate was pre-coated with the target antigen. Two of the wells were then aliquoted with 50μl of the negative and positive controls into the set wells, respectively. One well was left as the control (zero) blank. Similarly, we aliquoted 50μl appropriately diluted samples into the test sample wells with a dilution rate of 1/5. The solution was added at the bottom without touching the sidewalls of the well and the plate was shaken gently to mix the contents. The controls and test samples are incubated at 37°C for 30 minutes after sealed with a ready-made cover. After 30 minutes incubation, the cover was removed and the plate washed 5 times with buffer solution. Then 50μl of Horseradish Peroxidase (HRP) conjugate reagent was added to each well (except the blank well) and sealed again for incubation at 37°C for 30 minutes. The plates were then washed with buffer five times and aliquot 50μl of Tetramethyl benzidine (TMB) substrate A into each well, 50μl of TMB Substrate B added. Then plate was shaken gently by hand for 30 seconds, covered and incubated at 37°C for 15 minutes avoiding exposure to light. The HRP catalyzes TMB to produce a blue color product that changes to yellow after adding the acidic stop solution. The intensity of the yellow color is proportional to the YFV-IgG/CHIKV-IgG bound on the plate. The Optical density (OD) absorbance is measured spectrophotometrically at 450nm in a microplate reader, and the presence of YFV-IgG/ CHIKV-IgG is determined [35].

Each serum was also screened for IgG antibodies against ZIKV using the ZIKV NS1 BOB assay as previously developed in Nicaragua [36]. This is highly specific for ZIKV with minimal to no cross-reactivity to other flaviviruses. In brief, a 96 well plate is coated overnight with recombinant purified 1 ug/ml ZIKV NS1 Uganda strain (Native antigen) protein and blocked with PBS 1% bovine serum albumin for 1 h. The plate is then incubated with 1:10 diluted serum from each study participant for 1 hour and then HRP-labelled anti-ZIKV NS1 (mAB ZKA35, Absolute antibody) diluted 1:5000 in PBS with 1% BSA is added to each well and incubated for another 15 min. The plate is then washed and TMB substrate (Sigma) added to each well and incubated for 5–6 min in the dark and the reaction is stopped with 2N H_2_SO_4_ or 1N HCL. The plate is read on a plate reader (Multiskan^™^ FC Microplate Photometer) at an absorbance of 450 nm. Unconjugated ZKA35 rIgG1 (1:200 diluted) is added as a positive control and normal human serum (NHS) is added as a negative control (1:10 diluted). The percentage of ZKA35-HRP binding inhibition is calculated as described in detail by Balmaseda and his colleague [36]

The concentration of ZKA35-HRP used in the BOB assay corresponds to 70% of the maximal OD (450 nm) level as determined by interpolating a curve fitted with a 4-parameter nonlinear regression. A starting dilution at 1:10 and then 1:3 serial dilution of 12 points in assay diluent is performed using 50 μl ZKA35-HRP/well.

### Ethics approval and consent to participate

The study protocol was approved by the Institutional Review Board (IRB) of the Aklilu Lemma Institute of Pathobiology, Addis Ababa University. Permission to visit the study sites and to collect the blood samples was obtained from the Gambella Regional Health Office, district administration offices, and community leaders of each study site. The objective of the study was explained to each of the study participants and written consent was obtained from each participant. Finally, blood sample collection was carried out under aseptic conditions by experienced medical laboratory technicians.

### Statistical analysis

Collected data were coded and entered into Epi Data Software v.3.1 and analyzed using STATA 13.1. Socio-demographic characteristics were summarized using the frequencies and percentages while the seroprevalence of IgG antibodies elicited towards YFV, CHIKV, and ZIKV were calculated by dividing the number of participants with positive test results by the total number of study participants. The associations between the seroprevalence of IgG and the background characteristics such as age, sex, occupation, education, traveling/residency history in other countries or areas outside Gambella were assessed using the uni-variable logistic regression analysis. The effects of each independent variable (i.e. gender, age, occupation, ethnicity, education, and others) on the outcome variable after adjusting each independent variable for all other variables were analyzed using the multivariable logistic regression. The coefficient values of each independent variable (predictors) are included in the final reported model. All analysis results with a P-value below 0.05 were considered statistically significant.

## Results

### Socio-demographic characteristics of study participants

**Fig 2:**
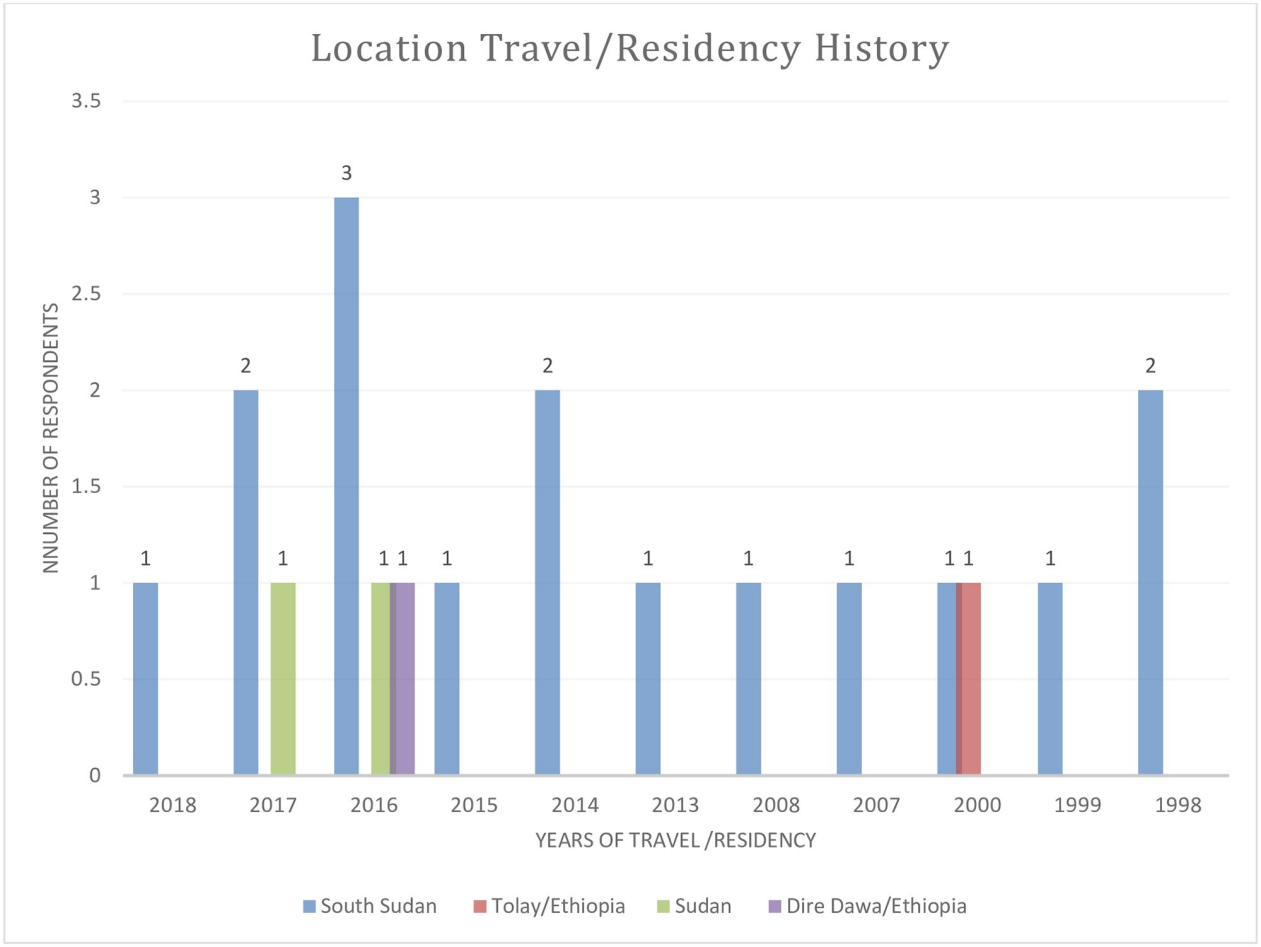
Traveling and residence out of Gambella region among those with a history of the travel.

The socio-demographic characteristics of all study participants are summarized in Table 1. A total of 150 individuals (96 males and 54 females, age ranging from 18–65 years) from two districts (33 from Lare and 117 from Itang special district) participated in this study. Most travelers were in the 18–30 age group (17.5%). The travel or living /residency history outside Gambella was higher in males (16.7%) than females (7.4%) as shown in Fig 2 (P-value>0.05). In the Lare district 15.2% of the participants had traveled or lived outside of Gambella (P-value >0.05). None of the study participants responded positively regarding YF vaccination.

**Table 1:** Socio-demographic characteristics (N = 150).

Variables	Response category	Travel history		History of working in the forest	
		Yes N(%)	No N(%)	Yes N(%)	No N(%)
Sex	Male	16(16.7)	80(83.3)	79(82.3)	17(17.7)
	Female	4(7.4)	50(92.6)	47(87.0)	7(13.0)
District	Itang	15(12.8)	102(87.2)	96(82.1)	21(17.9)
	Lare	5(15.2)	28(84.8)	30(90.9)	3(9.1)
Age (years)	18–30 years	10(17.5)	47(82.5)	48(84.2)	9(15.8)
	31–40 years	5(10.2)	44(89.8)	40(81.6)	9(18.4)
	≥41 years	5(11.4)	39(88.6)	38(86.4)	6(13.6)
Ethnicity	Nuer	15(15.5)	82(84.5)	92(94.8)	5(5.2)
	Agnewak	5(9.4)	48(90.6)	34(64.2)	19(35.8)
Education level	Informal	9(9.7)	84(90.3)	83(89.3)	10(10.7)
	Formal	11(19.3)	46(80.7)	43(75.4)	14(24.6)
Occupation	Pastoralist	8(13.8)	50(86.2)	53(91.4)	5(8.6)
	Agro pastoralist	6(16.7)	30(83.3)	35(97.2)	1(2.8)
	Others	6(10.7)	50(89.3)	38(67.9)	18(32.1)
History of vaccination	yes	0(0.0)	0(0.0)	-	-
	No	20 (13.3)	130(86.7)	126(84.0)	24(16.0)
History of bite by *Aedes* mosquito	Yes	17(14.4)	101(85.6)	105(89.0)	13(11.0)
	No	3(9.4)	29(86.6)	21(65.6)	11(34.4)

### Seroprevalence and independent predictors of sero-positivity for YFV infection

Out of the 135 randomly screened serum samples for IgG antibodies to YFV, only 4 samples (2.9%; 95% CI: 1.1–7.72%) were found to be positive (Table 2). All the YFV-IgG positive individuals were from the Itang district; hence site/district was omitted during logistic regression analysis due to zero outcomes from the Lare district. There were no identified risk factors significantly associated with seropositivity for specific IgG antibodies to YFV. However, there were higher proportion of males (3.6%) and individuals in the age group of 31–40 years (5%) having YFV specific IgG antibodies compared to the females (2%) or other age categories, respectively (Table 2). Participants with a history of residence/travel outside Gambella (AOR = 3.10, 95%CI: 0.21–44.56%) and those with an occupation engaged in agro-pastoralist (AOR = 1.92, 95% CI: 0.11–35.05%) were more likely to be IgG positive for YFV infection albeit not statistically significant. Participants with a history of a bite by *Ae* mosquitoes, and working in the forest showed protective odds in the contrary (Table 2).

**Table 2:** Sero-positivity for IgG antibody to YFV infection and associated factors (N = 135).

Variables	Category	YF IgG sero-status		COR (95% CI)	AOR (95% CI)
		Total tested(N)	Positive N (%)		
Sex	Male	84	3 (3.6)	1.85(0.19, 18.30)	1.32(0.10, 17.03)
	Female	51	1(2.0)	Ref	Ref
Age Group (years)	18–30	53	1(1.9)	0.79(0.05, 12.99)	0.77(0.03, 17.64)
	31–40	40	2(5.0)	2.16(0.19, 24.77)	2.29(0.17, 31.61)
	≥41	42	1(2.4)	Ref	Ref
Educational Status	Formal education	51	2(3.9)	1.7(0.23, 12.26)	1.31(0.10, 17.23)
	Informal education	84	2(2.4)	Ref	Ref
Occupation	pastoralist	55	1(1.8)	Ref	Ref
	Agro pastoralist	32	1(3.1)	1.74(0.10,28.84)	1.92(0.11, 35.05)
	Others	48	2(4.2)	2.35(0.21, 26.73)	1.18(0.05,25.41)
History of residence/travel outside Gambella	Yes	17	1 (5.9)	2.4(0.23, 24.45)	3.10(0.21, 44.56)
	No	118	3 (2.5)	Ref	Ref
History of working in the forest areas	Yes	113	3(2.6)	0.57(0.06, 5.77)	0.47(0.03, 7.68)
	No	22	1(4.5)	Ref	Ref
History of Bite by *Aedes* mosquito	Yes	109	3(2.7)	0.54(0.05, 5.44)	0.74(0.04, 12.25)
	No	26	1(3.85)	Ref	Ref
District	Itang	103	4(3.9)	-	-
	Lare	32	0(0.0)	-	-
History of vaccination	Yes	0(0.0)	0(0.0)		
	No	135	4(2.9)	-	-

CI (confidence interval), COR (crude odds ratio), and AOR (adjusted odds ratio), Ref (Reference category).

### Seroprevalence and independent predictors of sero-positivity for CHIKV infection

Out of the 90 samples 14 (15.6%, 95% CI: 9.3–24.8%) were positive for IgG antibodies against CHIKV (Table 3). In the multivariable logistic regression analysis model, a 14-fold lower seropositivity for CHIKV specific IgG was detected among pastoralists compared to agro-pastoral (AOR = 14.17; CI: 2.30, 87.30) (Table 3). A higher proportion of anti-CHIKV IgG antibodies was observed in the age group ≥41 compared to younger age categories although not statistically significant.

**Table 3:** Seropositivity for IgG antibody to CHIKV and associated factors (N = 90).

Variables	Category	CHIKV IgG sero-status		COR (95% CI)	AOR (95% CI)
		Total tested (N)	Positive N (%)		
Site	Itang	58	12(20.7)	Ref	Ref
	Lare	32	2(6.3)	0.25(0.05, 1.22)	0.43(0.06,2.82)
Sex	Male	53	12(22.6)	5.12(1.07, 24.46)*	3.78(0.58, 24.52)
	Female	37	2(5.4)	Reference	Reference
Age Group (years)	18–30	36	4(11.1)	0.48(0.12, 1.89)	0.75(0.11, 5.37)
	31–40	25	4(16.0)	0.73 (0.18, 2.95)	2.74(0.47, 16.08)
	≥41	29	6(20.7)	Ref	Ref
Educational Status	Informal education	64	8(12.5)	Ref	Ref
	Formal education	26	6(23.1)	2.1(0.65, 6.80)	2.64(0.36, 19.29)
Occupation	Pastoralist	49	2(4.1)	Ref	Ref
	Agro pastoralist	26	9(34.6)	12.44(2.44,63.47)*	14.17(2.30, 87.30)*
	Others	15	3(20.0)	5.88(0.88, 39.21)	2.20(0.17, 27.18)
History of residence outside Gambella	Yes	10	3(30.0)	2.68(0.60, 11.98)	2.44 (0.33, 17.69)
	No	80	11(13.7)	Ref	Ref
History of working in the forest areas	Yes	82	13(15.8)	1.32(0.14, 11.64)	0.90(0.05, 15.33)
	No	8	1(12.5)	Ref	Ref
History of Bite by *Aedes* mosquito	Yes	89	12(15.2)	0.54(0.10, 2.98)	0.73(0.1, 8.00)
	No	11	2(18.2)	Ref	Ref

CI (confidence interval), COR (crude odds ratio), AOR (adjusted odds ratio), and

*(significant at p<0.05), Ref (Reference category).

### Seroprevalence and independent predictors of sero-positivity for ZIKV infection

Out of the 150 samples tested for the presence of IgG antibodies against the ZIKV, 41 (27.3%; 95% CI: 20.7–35.1%) were found to be positive (Table 4). Multivariable logistic regression analysis showed that living in Lare (AOR = 11.5; 95% CI: 3.31–39.81%), being a female (AOR = 4.8; 95% CI: 1.62–14.63%) and being a pastoralist (AOR = 5.1; 95% CI: 1.44–17.80%) were significantly associated with seropositivity for ZIKV.

**Table 4:** Seropositivity for IgG antibody to ZIKV and associated factors in the study participants (N = 150).

Variables	Category	ZIKV IgG sero-status			COR (95% CI)	AOR (95% CI)
		Total tested N (%)	Positive N (%)	Equivocal N (%)		
Site	Itang	117	15(12.8)	2(1.7)	Ref	Ref
	Lare	33	26(18.8)	0(0.0)	21.8(8.20, 58.23)*	11.5(3.31, 39.81)*
Sex	Male	96	15(15.6)	1(1.0)	Ref	Ref
	Female	54	26(48.2)	1(1.8)	5.0(2.34, 10.65)*	4.8(1.62, 14.63)*
Age Group (years)	18–30	57	23(40.3)	0(0.0)	2.1(0.90,4.8)	1.5(0.39, 5.61)
	31–40	49	11(22.5)	1(2.0)	1.5(0.53, 4.0)	0.80(0.21,3.13)
	≥41	44	7(15.9)	1(2.3)	Ref	Ref
Educational Status	Informal education	93	32(34.4)	1(1.1)	2.59(1.16, 5.76)*	1.0(0.28,3.68)
	Formal education	57	9(15.8)	1(1.8)	Ref	Ref
Occupation	Pastoralist	58	32(55.2)	1(1.7)	8.2(2.78, 24.1)*	5.1(1.44, 17.80)*
	Others	56	5(8.9)	0(0.0)	0.61(0.16, 2.27)	1.5(0.27,8.22)
	Agro pastoralist	36	4(11.1)	1(2.8)	Ref	Ref
History of residence /travel outside Gambella	Yes	20	6(30.0)	0(0.0)	1.10(0.38, 3.0)	1.4(0.29,6.65)
	No	130	35(26.9)	2(1.5)	Ref	Ref
History of working in the forest areas	Yes	126	36 (28.6)	2(1.6)	1.64(0.57, 4.72)	1.15(0.20, 6.49)
	No	24	5(20.8)	0(0.0)	Ref	Ref
History of bite by *Aedese* mosquito	Yes	118	36(30.5)	2(1.7)	5.7(1.28, 25.37)*	1.34(0.21, 8.47)
	No	32	5(15.6)	0(0.0)	Ref	Ref

CI (confidence interval), COR (crude odds ratio), AOR (adjusted odds ratio), and

*(significant at p<0.05), Ref (Reference category).

## Discussion

Arboviral infections and outbreaks in Ethiopia have been documented since the 1960s, yet the present epidemiological situation in the country remains unknown [37]. This is the first sero-epidemiological study of YFV, CHIKV, and ZIKV infections in community members from the Gambella regional state of Ethiopia.

Our study examines the community-based sero-epidemiology of YFV, CHIKV, and ZIKV in two selected districts of the Gambella region in South West Ethiopia. Previously a few studies on arboviruses have been conducted in other regions of Ethiopia and were mainly part of outbreak investigations at that time including dengue fever (DF) and YF [3, 19, 22, 37, 38].

We found a seroprevalence of 2.9% of YFV specific-IgG in collected blood samples.

Of the four YFV IgG-positive individuals, three had no history of residency or travel outside the Gambella region, thus indicating that YFV infections occurred in this area.

Besides, three of the four individuals reported a history of working in the forest area for farming, where they might have been exposed to mosquito bites. Indeed arboviruses commonly circulate in the forest area in a sylvatic cycle involving primates as reservoir hosts [13]. The prevalence rate of IgG antibodies against YFV in the Gambella region is in line with the seroprevalence detected in a previous study conducted in Djibouti (14 ELISA positive out of 903 screened) [39]. However, another study from another part of Ethiopia revealed that the seroprevalence of YFV among study participants was 0.6% in pooled samples [19]. The slightly higher seroprevalence that we found in the Gambella region could be explained by the higher rate of daily commuters from neighboring countries (South Sudan, Sudan), migration of refugees, and seasonal movement of pastoralists from North and West Africa in search of pasture. Our findings show a lower anti-YFV IgG antibody response compared to other seroprevalence studies conducted in South Omo, Ethiopia [22] where the authors found 49.5% seropositivity for YFV IgG antibodies. A higher seroprevalence of YFV IgG antibodies was also reported from the Borena area of Southern Ethiopia (12.5% of participants) [40], and Kenya (6% of participants) [41]. These variations might be due to different sample sizes, and higher vaccination history in the population. No YF vaccinations was recorded in any of our subjects at both study sites. The comparison of different studies is difficult due to differences in methodology, diagnostic tools, and characteristics of the study populations. For instance, the current study focused on the detection of IgG antibodies in blood samples of healthy donors using an ELISA while others implemented advanced diagnosis methods. In addition, other groups used sera from individuals with suspected infection rather than from an apparently healthy community. IgG antibodies against YFV were also observed in a study conducted in Kenya among subjects between 30–40 years of age [42].

Among the 14 individuals having anti-CHIKV IgG, only three had a history of residency or travel out of Gambella. This might indicate that CHIKV infections occurred in the study area but detection of IgM or viral RNA is necessary to confirm current virus circulation. Almost all seropositive individuals were engaged in farming activities in the forest area and had reported mosquito bites. The prevalence of anti-CHIKV IgG in our study (seroprevalence 15.6%) is lower than in Sudan, Kassala (73.1%) [43], where only patients suffering from unknown fever were tested. In contrast, Khartoum state in Sudan and Southern Mozambique reported a low seroprevalence of CHIKV IgG antibodies of 2.2% [44] and 4.3% [45], respectively among febrile patients. Thus, these studies differ from the present study in Gambella, where we screened only healthy subjects. The majority of the CHIKV IgG+ cases in our study were between 31–40 years of age, which is similar to findings of a seroprevalence study in Tanzania [46]. The highest IgG seroprevalence of CHIKV was found in males which was fivefold (22.6%) higher compared to females, supporting the contention that males are more exposed to mosquito bites during farming activity or other similar travel or working related factors. These findings are in agreement with another study that showed that males are more susceptible than females [47, 48]. However, other seroprevalence studies observed the opposite trend, where females were more often CHIKV IgG+ compared to males [49, 50]. The detection of IgG against CHIKV was significantly associated with the agro-pastoralist lifestyle compared to a pastoralist one, which could be associated with the job-related risk including environmental suitability for vector and maintenance of the virus in such conditions as well as the intimate contact between humans, and primates. The discrepancy with results from other studies might be due to sampling size and proportions of the various categories such as gender and age. Furthermore, the participating community could have various habits, behaviors, occupations, traditions, and local practices that would expose them to the virus more or less likely.

In the current study, there is serological evidence of human exposure to ZIKV in the two districts of the Gambella region, Southwest Ethiopia. Antibodies against ZIKV were found in 41 individuals (27.3%; 95% CI: 20.7, 35.1%). Among those 41, only six individuals reported a history of traveling or residing outside of Gambella. Almost all individuals reported visiting forest areas for their farming activities, where they might have been infected via mosquitoes which are in in close contact with the reservoir hosts such as primates. Indeed, most of the positive individuals had reported mosquito bites. Markedly, the seroprevalence of anti- ZIKV IgG found in Gambella was higher than studies conducted elsewhere in Ethiopia in 2018 (0.4%) [19], in Kenya (3.9%) [43], and Zambia (6.1%) [51], but was similar to the seroprevalence reported in Nigeria (31%) [52]. Our study confirms that ZIKV infections have occurred in the study area of Ethiopia. A large fraction of the ZIKV IgG+ cases were found in the Lare district (11 fold higher than Itang special district), which could be due to Lare being the main entry point from South Sudan to Ethiopia, and is a temporary residential location and a registration place for refugees from South Sudan. Neighboring countries such as Kenya have also reported a high seroprevalence of ZIKV IgG+ antibodies in many region [53, 54]. Significantly higher ZIKV IgG+ antibodies were found in females in this study which is similar to a study conducted elsewhere [55]. Studies indicated that females in the sexually active age group are more likely to get ZIKV than males; sexual transmission is the most probable cause [56]. A gender-specific prevalence of ZIKV is also observed in mouse models [57] as female mice are more affected. In contrast to this study’s finding, a high risk of ZIKV infection in males was observed in Kenya [42] even if not significant. These differences might be explained by the variation in gender proportion, the difference in laboratory tests applied, presence of non-vector transmissions, and the traditions of the community which poses a predisposition factor for ZIKV infection. In general, a large fraction of the age groups 18–30 and 31–40 years was ZIKV IgG+ compared to individuals in the age group ≥41 years, which is in line with a study result from Zambia where study participants between 24–44 years of age showed a higher trend of ZIKV IgG seropositivity compared to older participants [51]. This result could be related to differences in mobility, occupation, and immune status between age groups. Participants with a history of residence or travel outside of Gambella also showed a higher trend for being ZIKV IgG+ which is in agreement with other study results [51, 58]. Participants who had a history of working in the forest areas were more likely to be ZIKV IgG+. Notably, pastoralists were found to be significantly more affected compared to agro-pastoralists which is in line with another study from Kenya [42]. This could be due to the tradition of the pastoralists and their lifestyle associated with long-distance movements with their livestock in search of pasture or water which exposes them more to forest areas and mosquito breeding sites close to water sources. Participants with informal education were more likely to have ZIKA IgG+ antibodies relative to those who have attended formal education. This might correlate with lower knowledge of disease transmission and the source of infection as shown by a study conducted in Nigeria [59].

### Limitation of the study

One limitation of the study is that we did not use confirmatory tests such as Plaque Reduction Neutralization Test (PRNT) as proof of the viral agent due to budget limitations. In addition, we were unable to screen all the serum samples for YFV and CHIKV also due to budget limitations. Thus, the number of serum samples screened may be a limitation of the study. Finally, we did not conduct IgM ELISA screening to help distinguish recent exposure vs. more distant viral infection.

## Conclusions

Our community-based seroprevalence study showed the circulation of YFV, CHIKV, and ZIKV in the two districts of the Gambella region of Ethiopia. We found that the seroprevalence of anti-CHIKV and anti-ZIKV IgG antibodies were significantly higher in the agro-pastoral and pastoralists communities respectively. Moreover, the seroprevalence of anti-ZIKV IgG was significantly higher in females and in individuals from the Lare district. Therefore, additional testing options are needed in local health facilities and laboratories to help implement mosquito-borne viral disease prevention and control programs.

## Supporting information

S1 Protocol (DOCX)

S1 Questionnaire (DOCX)

S2 Questionnaire (DOCX)

S1 Data (XLS)

## References

[1] CDC. Why is Dengue a Global Issue? [Internet]. 2020 [cited 2020 Oct 29]. https://www.cdc.gov/dengue/training/cme/ccm/page51440.html

[2] Gould EA, Higgs S. Impact of climate change and other factors on emerging arbovirus diseases. Trans R Soc Trop Med Hyg. 2009;103(2):109–21, doi: 10.1016/j.trstmh.2008.07.025 18799177 PMC2915563

[3] Lilay A, Asamene N, Bekele A, Mengesha M, Wendabeku M, Tareke I, et al. Reemergence of yellow fever in Ethiopia after 50 years, 2013: epidemiological and entomological investigations. BMC Infect Dis. 2017;17(1):343. doi: 10.1186/s12879-017-2435-4 28506254 PMC5432991

[4] Gould, Pettersson J, Higgs S, Charrel R, de Lamballerie X. Emerging arboviruses: Why today? One Heal. 2017;4(April):1–13. doi: 10.1016/j.onehlt.2017.06.001 28785601 PMC5501887

[5] Gould EA, Solomon T. Pathogenic flaviviruse. Lancet. 2008;371:500–509. doi: 10.1016/S0140-6736(08)60238-X 18262042

[6] Wachtman L, Keith M. Viral Diseases of Nonhuman Primates. Second Edi. Abee CR, Keith M, Tardif S, Morris T, editors. Vol. 2, in Nonhuman Primates in Biomedical Research. Elsevier Inc.; 2012.

[7] NCDC NC for DC. Situation Report: Yellow Fever Outbreak in Nigeria (NCDC, Jabi Abuja, Nigeria). 2018.

[8] WHO. Yellow Fever Situation Report (WHO, Geneva, Switzerland). 2016;

[9] WHO/AFRO. The yellow fever outbreak in Angola and Democratic Republic of the Congo ends (2017). http://www.who.int/csr/disease/yellowfev/en/. 2017;

[10] Sérié C, Andral L, Poirier A, Lindrec A, Neri P. Etudes sur la fièvre jaune en Ethiopie. 6. Etude épidémiologique. Bull World Health Organ. 1968;38(6):879–84. 5303663 PMC2554520

[11] WHO. Yellow Fever-Ethiopia. Emergencies preparedness, response Disease Outbreak News (DONs) [Internet]. 2020 [cited 2020 Oct 21]. p. 22 April 2020. https://www.who.int/csr/don/22-april-2020-yellow-fever-ethiopia/en/

[12] Robinson M. An epidemic of virus disease in Southern Province, Tanganyika Territory, in 1952–53–1: Clinical features. Trans R Soc Trop Med Hyg. 1955;49:28–32. doi: 10.1016/0035-9203(55)90080-8 14373834

[13] Thiberville S, Moyen N, Dupuis-Maguiraga L, Nougairede A et al. Chikungunya fever: epidemiology, clinical syndrome, pathogenesis and therapy. Antivir Res. 2013;99(3):345–70. doi: 10.1016/j.antiviral.2013.06.009 23811281 PMC7114207

[14] Anyangu AS, Gould LH, Sharif SK, Nguku PM, Omolo JO, Mutonga D, et al. Risk factors for severe Rift Valley fever infection in Kenya, 2007. Am J Trop Med Hyg. 2010;83(2 Suppl):14–21. doi: 10.4269/ajtmh.2010.09-0293 20682901 PMC2913492

[15] Inziani M, Adungo F, Awando J, Kihoro R, Inoue S, Morita K, et al. Seroprevalence of yellow fever, dengue, West Nile and chikungunya viruses in children in Teso South Sub-County, Western Kenya. Int J Infect Dis [Internet]. 2020;91:104–10. Available from: 10.1016/j.ijid.2019.11.004 31712089

[16] Chipwaza B, Mugasa J, Selemani M, Amuri M, Mosha F, Ngatunga S et al. Dengue and Chikungunya fever among viral diseases in outpatient febrile children in Kilosa district hospital, Tanzania. PLoS Negl Trop Dis. 2014;Nov; 8(11). doi: 10.1371/journal.pntd.0003335 25412076 PMC4239002

[17] Clements TL, Rossi CA, Irish AK, Kibuuka H, Eller LA, Robb ML, et al. Chikungunya and o’nyong-nyong viruses in Uganda: Implications for diagnostics. Open Forum Infect Dis. 2019;6(3):1–7. doi: 10.1093/ofid/ofz001 31660384 PMC6411207

[18] Konongoi S, Nyunja A, Ofula V, Owaka S, Koka H, Koskei E, et al. Human and entomologic investigations of chikungunya outbreak in Mandera, Northeastern Kenya, 2016. PLoS One. 2018;13(10). 10.1371/journal.pone.0205058 PMC6181335 30308064

[19] Mengesha Tsegaye M, Beyene B, Ayele W, Abebe A, Tareke I, Sall A, et al. Seroprevalence of yellow fever and related Flavi viruses in Ethiopia: A public health perspective. BMC Public Health. 2018;18(1):1–10. doi: 10.1186/s12889-018-5726-9 30107830 PMC6092792

[20] Alayu M, Teshome T, Amare H, Kinde S, Belay D, Assefa Z. Risk Factors for Chikungunya Outbreak in Kebridhar City, Somali Ethiopia, 2019. Unmatched Case-Control Study. 2020;

[21] Geleta D, Tesfaye N, Ayigegn H. Epidemiological Description of Chikungunya Virus Outbreak in Dire Dawa Administrative City, Western Ethiopia, 2019. Int J Clin Exp Med Sci. 2020;6(3):41.

[22] Endale A, Michlmayr D, Abegaz WE, Asebe G, Larrick JW, Medhin G, et al. Community-based seroprevalence of chikungunya and yellow fever in the South Omo Valley of Southern Ethiopia. 2020;1–16. Available from: 10.1371/journal.pntd.0008549 PMC7470273 32881913

[23] Dick G, Kitchen S, Haddow A. Zika virus. I. Isolations and serological specificity. Trans R Soc Trop Med Hyg. 1952;46:509–20. doi: 10.1016/0035-9203(52)90042-4 12995440

[24] Smithburn K. Neutralizing antibodies against certain recently isolated viruses in the sera of human beings residing in East Africa. J Immunol. 1952;69:223–34. 14946416

[25] Hennessey M, Fischer M, Staples J. Zika Virus Spreads to New Areas—Region of the Americas, May 2015-January 2016. MMWR Morb Mortal Wkly Rep 65. 2016. doi: 10.15585/mmwr.mm6503e1 26820163

[26] Musso D, Beltrame A, Zammarchi L, Gianluca Zuglian FG, Andrea Angheben VM, Degani Monica, et al. Zika Virus Transmission from French Polynesia to Brazil. 2016;21(10):2015. 10.3201/eid2110.151125 PMC4593458 26403318

[27] Mysorekar IU. Zika Virus Takes a Transplacental Route to Infect Fetuses: Insights from an Animal Model. 2017;(June):168–70. PMC6140214 30228574

[28] Atoni E, Waruhiu C, Nganga S, Xia H, Yuan Z. Arboviruses of Human Health significance in Kenya Atoni. African J Heal Sci. 2018;31(1):122–41.

[29] Markoff L. Yellow fever outbreak in Sudan. N Engl J Med. 2013;368(8):689–91. doi: 10.1056/NEJMp1300772 23387798

[30] WHO. South Sudan declares Rift Valley fever outbreak in parts of Eastern Lakes State. World Health Organization (WHO) report Published on12 Mar 2018. 2018.

[31] Woube M. Flooding and sustainable land–water management in the lower Baro–Akobo river basin, Ethiopia. App Geogr. 1999;19:235–51.

[32] Central Statistical Agency. Population Projections for Ethiopia 2007–2037. 2013.

[33] Chauhan RP, Dessie ZG, Noreddin A, El Zowalaty ME. Systematic review of important viral diseases in africa in light of the ‘one health’ concept. Pathogens. 2020;9(4). doi: 10.3390/pathogens9040301 32325980 PMC7238228

[34] Onyango C., Ofula V., Sang R., Konongoi S., Sow A, De Cock KM, et al. Yellow fever outbreak, Imatong, southern Sudan. Emerg Infect Dis. 2004;10(6):1063–8. doi: 10.3201/eid1006.030738 15207058 PMC3323161

[35] Abbexa Ltd. www.abbexa.com [Internet]. 2019 [cited 2019 Sep 20]. https://www.abbexa.com/index.php?route=product/search&search=yellowfever

[36] Balmaseda A, Stettler K, Medialdea-Carrera R, Collado D, Jin X, Zambrana JV, et al. Antibody-based assay discriminates Zika virus infection from other flaviviruses. Proc Natl Acad Sci U S A. 2017;114(31):8384–9. doi: 10.1073/pnas.1704984114 28716913 PMC5547631

[37] Ardoin P, Rodhain F, Hannoun C. Epidemiologic study of arboviruses in the Arba-Minch district of Ethiopia. Trop Geogr Med. 1976;28(4):309–15. 13526

[38] Woyessa A, Mengesha M, Kassa W, Kifle E, Wondabeku M, Girmay A. The first acute febrile illness investigation associated with dengue fever in Ethiopia, 2013: A descriptive analysis. Ethiop J Heal Dev. 2013;28(3):155–61.

[39] Andayi F, Charrel RN, Kieffer A, Richet H, Pastorino B, Leparc-Goffart I, et al. A Sero-epidemiological Study of Arboviral Fevers in Djibouti, Horn of Africa. PLoS Negl Trop Dis. 2014;8(12). doi: 10.1371/journal.pntd.0003299 25502692 PMC4263616

[40] Nigussie E, Shimelis T, Eshetu D, Shumie G, Chali W, Aseffa A, et al. Original Article Seropositivity of Yellow Fever Virus Among Acute Febrile Pa- Tients Attending Selected Health Facilities in Borena District,. 2020;58(1):57–62.

[41] Kwallah AO, Inoue S, Thairu-Muigai AW, Kuttoh N, Morita K, Mwau M. Seroprevalence of yellow fever virus in selected health facilities in Western Kenya from 2010 to 2012. Jpn J Infect Dis. 2015;68(3):230–4. doi: 10.7883/yoken.JJID.2014.288 25672346

[42] Chepkorir E, Tchouassi DP, Konongoi SL, Lutomiah J, Tigoi C, Irura Z, et al. Serological evidence of Flavivirus circulation in human populations in Northern Kenya: An assessment of disease risk 2016–2017. Virol J. 2019;16(1):1–10. 31101058 10.1186/s12985-019-1176-y PMC6525424

[43] Mohamed N, Magzoub M, Mohamed REH, Aleanizy FS, Alqahtani FY, Nour BYM, et al. Prevalence and identification of arthropod-transmitted viruses in Kassala state, Eastern Sudan. Libyan J Med [Internet]. 2019;14(1). Available from: 10.1080/19932820.2018.1564511 30716013 PMC6366427

[44] Alfadil MF, Rahama ABM, Bakheit AM, Ibrahim MEA, Abdullah HM, Yassin ME. Seroprevalence of chikungunya virus with high pyrexia patients in Khartoum state, Sudan Introduction: LMJ. 2017;3(1):24–8.

[45] Gudo E, Pinto G, Vene S, Mandlaze A, Muianga A, Cliff J, et al. Serological Evidence of Chikungunya Virus among Acute Febrile Patients in Southern Mozambique. PLoS Negl Trop Dis. 2015;9(10). doi: 10.1371/journal.pntd.0004146 26473605 PMC4608817

[46] Kajeguka D., Kaaya R., Mwakalinga S, Ndossi R, Ndaro A, Chilongola J., et al. Prevalence of dengue and chikungunya virus infections in north-eastern Tanzania: A cross sectional study among participants presenting with malaria-like symptoms. BMC Infect Dis. 2016;16(1):1–9. doi: 10.1186/s12879-016-1511-5 27112553 PMC4845349

[47] Azami NAM, Salleh SA, Shah SA, min Neoh H, Othman Z, Zakaria SZS, et al. Emergence of chikungunya seropositivity in healthy Malaysian adults residing in outbreak-free locations: Chikungunya seroprevalence results from the Malaysian Cohort. BMC Infect Dis. 2013;13(1): doi: 10.1186/1471-2334-13-67 23379541 PMC3651385

[48] Sissoko D, Moendandze A, Malvy D, Giry C, Ezzedine K, Solet J., et al. Seroprevalence and risk factors of chikungunya virus infection in Mayotte, Indian Ocean, 2005–2006: A populationbased survey. PLoS One. 2008;3(8):e3066. doi: 10.1371/journal.pone.0003066 18725980 PMC2518850

[49] Kawle AP, Nayak AR, Bhullar SS, Borkar SR, Patankar SD, Daginawala HF, et al. Seroprevalence and clinical manifestations of chikungunya virus infection in rural areas of Chandrapur, Maharashtra, India. J Vector Borne Dis. 2017;54(1):35–43. 28352044

[50] Mohanty I, Dash M, Sahu S, Narasimham M, Panda P, Padhi S. Seroprevalence of chikungunya in southern Odisha. J Fam Med Prim Care. 2013;2(1):33–6. doi: 10.4103/2249-4863.109939 24479040 PMC3894024

[51] Babaniyi OA, Mwaba P, Songolo P, Mazaba-Liwewe ML, Mweene-Ndumba I, Masaninga F, et al. Seroprevalence of Zika virus infection specific IgG in Western and North-Western Provinces of Zambia. 2015;(January). 10.4314/ahs.v15i3.14 PMC4765448 26957968

[52] Fagbami A. Zika virus infections in Nigeria: virological and seroepidemiological investigations in Oyo State. JHyg(Lond). 1979;83(2):213–9. doi: 10.1017/s0022172400025997 489960 PMC2129900

[53] LaBeaud AD, Muchiri EM, Ndzovu M, Mwanje MT, Muiruri S, Peters CJ, et al. Interepidemic Rift Valley fever virus seropositivity, northeastern Kenya. Emerg Infect Dis. 2008;14(8):1240–1246. doi: 10.3201/eid1408.080082 18680647 PMC2600406

[54] Sutherland LJ, Cash AA, Huang YJS, Sang RC, Malhotra I, Moormann AM, et al. Serologic evidence of arboviral infections among humans in Kenya. Am J Trop Med Hyg. 2011;85(1):158–61. doi: 10.4269/ajtmh.2011.10-0203 21734142 PMC3122361

[55] Victor J, Bustos F, Burger-calderon R, Collado D, Sanchez N, Ojeda S, et al. Seroprevalence, risk factor, and spatial analyses of Zika virus infection after the 2016 epidemic in. 2018;115(37). 10.1073/pnas.1804672115 PMC6140532 30150394

[56] Coelho FC, Durovni B, Saraceni V, Lemos C, Codeco CT, Camargo S, et al. Higher incidence of Zika in adult women than adult men in Rio de Janeiro suggests a significant contribution of sexual transmission from men to women. Int J Infect Dis. 2016 Oct 1;51:128–32. doi: 10.1016/j.ijid.2016.08.023 27664930

[57] Duggal NK, Mcdonald EM, Ritter JM, Brault AC. Sexual transmission of Zika virus enhances in utero transmission in a mouse model OPEN. Sci REpORTS | [Internet]. 2018;8:4510. Available from: www.nature.com/scientificreports/10.1038/s41598-018-22840-6 PMC5852059 29540804

[58] Porse CC, Messenger S, Vugia DJ, Jilek W, Salas M, Watt J, et al. Travel-associated zika cases and threat of local transmission during global outbreak, California, USA. Emerg Infect Dis. 2018;24(9):1626–32. doi: 10.3201/eid2409.180203 30124194 PMC6106427

[59] Ndibuagu EO. Formal Education Related Pattern of Awareness and Basic Knowledge on Zika Virus Disease, among Women Visiting Children Immunization Unit in a Tertiary Hospital, Southeast Nigeria. Health (Irvine Calif). 2018;10(11):1576–96.

