# Supplementary material for: Seroprevalence of Yellow fever, Chikungunya, and Zika virus at a community level in the Gambella Region, South West Ethiopia
Source: PLoS One. 2021 Jul 8;16(7):e0253953. doi: 10.1371/journal.pone.0253953 (PMC8266044; doi:10.1371/journal.pone.0253953)
Supplement: S1 Protocol — (DOCX) [file pone.0253953.s001.docx]

# Human Yellow Fever Virus IgG (YFV-IgG) ELISA kit

Range: Qualitative

Sensitivity: Qualitative

Application: For qualitative detection of YFV-IgG in human serum, plasma, Tissue Homogenaates and cell culture supernatants.

**Principe of the Assay**

A 96 well plate has been pre-coated with the target antigen. Controls or test samples are added to the appropriate wells and incubated. Free components are washed away with wash buffer. HRP conjugated detection reagents are used to visualize HRP enzymatic reaction. TMB is catalyzed by HRP to produce a blue colour product that changes to yellow after adding acidic stop solution. The intensity of the color yellow is proportional to the YFV-IgG amount bound on the plate. The O.D. absorbance is measured spectrophotometrically at 450nm in a microplate reader, and the presence of YFV-IgG can be determined.

**Materials used (for 96 well)**

**Kit components**

- 1. one pre-coated 96-well microplate ((8*12 well strips)),
  2. Positive control 0.5ml,
  3. Negative control: 0.5ml
  4. Wash buffer (30X): 20ml. dilution: 1:30
  5. Sample diluent buffer: 6ml
  6. HRP conjugate reagent (RTU) : 6ml
  7. Stop solution: 6ml
  8. TMB substrate A: 6ml
  9. TMB substate B: 6ml
  10. Plate sealer: 2
  11. Hermetic bag: 1

Materials used

1. 37^0^C incubater
2. Microplate reader (Wavelength 450 nm)
3. Multi and Single channel pipette and sterile pipette tips
4. Squirt bottle or automated microplate washer
5. ELISA shaker
6. 1.5ml tubes
7. Deionized or distilled water
8. Absorbent filter papers
9. 100 ml and 1 liter graduated cylinders
10. **Preparation of samples and reagents**

Isolates the test samples soon after collecting and analyze immediately at 1/5 dilution (within 2 hours) or aliquot and store at -20^0^C or -80^0^C for long-term storage.

1. **Sample**

The serum samples should be collected into a serum separator tube. Coagulate the serum by leaving the tube undisturbed in a vertical position overnight at 4^0^C or at room temperature for up to 60 minutes. Centrifuge at approximately 1000Xg for 20 minutes. Analyze the serum immediately or aliquot and store at -20^0^C or -80^0^C. (The storage of samples should be undiluted. Once ready to analyze, thaw samples and dilute 1/5).

1. **Wash buffer**

Dilute the concentrated Wash buffer 30-fold (1/30) with distilled water (i.e add 20 ml of concentrated wash buffer into 580ml of distilled water).

1. **Assay procedure**

Equilibrate the kit components and samples to room temperature prior to use.

1. Set positive/negative controls, test sample and control(zero/blank) wells on the pre-coated plate and record their positions.
2. Aliquot 50µl of the negative and positive controls into the set wells. Leave one well as the control (zero) blank well.
3. Aliquot 50µl of appropriately diluted samples into the test sample wells. Samples should be diluted 1/5. Add the solution at the bottom without touching the sidewalls of the well. Shake the plate gently to mix the contents.
4. Seal the plate with a cover and incubate at 37^0^C for 30 minutes
5. Remove the cover and discard the solution. Wash the plate 5 times with 1X wash buffer. Fill each well completely with Wash buffer (300µl) using a multi-channel Pipette or auto washer (1-2 minute soaking period is recommended). Complete removal of liquid at each step is essential for good performance. After the final wash, remove any remaining Wash Buffer by aspirating or decanting. Invert the plate and blot it against clean absorbent paper towels.
6. Add 50µl of HRP conjugate reagent into each well (except the blank well). Add the solution at the bottom of each well without touching the sidewall.
7. Seal the plate with a cover and incubate at 37^0^C for 30 minutes.
8. Remove the cover and repeat the aspiration/wash process 5 times as explained in step 5.
9. Aliquot 50µl of TMB substrate A into each well, and then add 50µl of TMB Substrate B. Vortex the plate gently on an ELISA shaker for 30 seconds (Or shake gently by hand for 30 seconds). Cover the plate and incubate at 37^0^C for 15 minutes. Avoid exposure to light.
10. Add 50µl of Stop solution into each well to stop the enzyme. It is important that the Stop solution be mixed quickly and uniformly throughout the microplate to inactivate the enzyme completely.
11. Ensure that there are no fingerprints or water on the bottom of the plate, and that the fluid in the wells is free of bubbles. Measure the absorbance at 450 nm immediately.
12. **Analysis**
13. **Calculations**:

Mean absorbance of the positive control should be ≥1.00

Mean absorbance of the negative control should be ≤0.10

CUT OFF value=Negative control + 0.15

1. **Interpretation of results**:

If the positive control value is ≥ 1.00 and the negative control value is ≤ 0.10, the test is valid, otherwise, the test is invalid.

If O.D. of samples < CUT OFF, the test samples are considered negative.

If O.D. of samples ≥ CUT OFF, the test samples are considered positive.

1. **Precautions**
2. Before using the kit, centrifuge the **tubes** to bring down the contents trapped in the lid
3. **Avoid foaming** or **bubbles** when **mixing** or **reconstituting** components.
4. If crystals have formed in the concentrated **Wash Buffer**, warm to room temperature and mix gently until the crystals have completely **dissolved.**
5. It is recommended measuring each controls and samples in duplicate.
6. Do not let the wells uncovered for extended periods between incubation. Once reagents are added to the wells do not let the strips dry as this can inactivate the biological material on the plate. Incubation time and temperature must be controlled.
7. Ensure plates are properly sealed or covered during incubation steps.
8. Complete removal of all solutions and buffers during wash steps is necessary for accurate measurement readings
9. Do not reuse pipette tips and tubes to avoid cross contamination.
10. Do not use expired components from different kit.
11. The TMB Substrate B is easily contaminated: protect from light and work under sterile conditions when handling the TMB substrate solution. Equilibrate at room temperature prior to use. Unreacted substrate should be colorless or very yellow in appearance. Aspirate the dosage needed with sterilized tips and do not dump the residual solution back into the vial.

# Human Chikungunya Virus IgG (CHIKV-IgG) ELISA kit

Range: Qualitative

Sensitivity: Qualitative

Application: For qualitative detection of CHIKV-IgG in human serum, plasma, Tissue Homogenaates and cell culture supernatants.

**Principe of the Assay**

A 96 well plate has been pre-coated with the target antigen. Controls or test samples are added to the appropriate wells and incubated. Free components are washed away with wash buffer. HRP conjugated detection reagents are used to visualize HRP enzymatic reaction. TMB is catalyzed by HRP to produce a blue colour product that changes to yellow after adding acidic stop solution. The intensity of the color yellow is proportional to the CHIKV-IgG amount bound on the plate. The O.D. absorbance is measured spectrophotometrically at 450nm in a microplate reader, and the presence of CHIKV-IgG can be determined.

**Materials used (for 96 well)**

**Kit components**

1. one pre-coated 96-well microplate ((8*12 well strips)),
2. Positive control 0.5ml,
3. Negative control: 0.5ml
4. Wash buffer (30X): 20ml. dilution: 1:30
5. Sample diluent buffer: 6ml
6. HRP conjugate reagent (RTU) : 6ml
7. Stop solution: 6ml
8. TMB substrate A: 6ml
9. TMB substate B: 6ml
10. Plate sealer: 2
11. Hermetic bag: 1

Materials used

1. 37^0^C incubater
2. Microplate reader (Wavelength 450 nm)
3. Multi and Single channel pipette and sterile pipette tips
4. Squirt bottle or automated microplate washer
5. ELISA shaker
6. 1.5ml tubes
7. Deionized or distilled water
8. Absorbent filter papers
9. 100 ml and 1 liter graduated cylinders
10. **Preparation of samples and reagents**

Isolates the test samples soon after collecting and analyze immediately at 1/5 dilution (within 2 hours) or aliquot and store at -20^0^C or -80^0^C for long-term storage.

1. **Sample**

The serum samples should be collected into a serum separator tube. Coagulate the serum by leaving the tube undisturbed in a vertical position overnight at 4^0^C or at room temperature for up to 60 minutes. Centrifuge at approximately 1000Xg for 20 minutes. Analyze the serum immediately or aliquot and store at -20^0^C or -80^0^C. (The storage of samples should be undiluted. Once ready to analyze, thaw samples and dilute 1/5).

1. **Wash buffer**

Dilute the concentrated Wash buffer 30-fold (1/30) with distilled water (i.e add 20 ml of concentrated wash buffer into 580ml of distilled water).

1. **Assay procedure**

Equilibrate the kit components and samples to room temperature prior to use.

1. Set positive/negative controls, test sample and control(zero/blank) wells on the pre-coated plate and record their positions.
2. Aliquot 50µl of the negative and positive controls into the set wells. Leave one well as the control (zero) blank well.
3. Aliquot 50µl of appropriately diluted samples into the test sample wells. Samples should be diluted 1/5. Add the solution at the bottom without touching the sidewalls of the well. Shake the plate gently to mix the contents.
4. Seal the plate with a cover and incubate at 37^0^C for 30 minutes
5. Remove the cover and discard the solution. Wash the plate 5 times with 1X wash buffer. Fill each well completely with Wash buffer (300µl) using a multi-channel Pipette or auto washer (1-2 minute soaking period is recommended). Complete removal of liquid at each step is essential for good performance. After the final wash, remove any remaining Wash Buffer by aspirating or decanting. Invert the plate and blot it against clean absorbent paper towels.
6. Add 50µl of HRP conjugate reagent into each well (except the blank well). Add the solution at the bottom of each well without touching the sidewall.
7. Seal the plate with a cover and incubate at 37^0^C for 30 minutes.
8. Remove the cover and repeat the aspiration/wash process 5 times as explained in step 5.
9. Aliquot 50µl of TMB substrate A into each well, and then add 50µl of TMB Substrate B. Vortex the plate gently on an ELISA shaker for 30 seconds (Or shake gently by hand for 30 seconds). Cover the plate and incubate at 37^0^C for 15 minutes. Avoid exposure to light.
10. Add 50µl of Stop solution into each well to stop the enzyme. It is important that the Stop solution be mixed quickly and uniformly throughout the microplate to inactivate the enzyme completely.
11. Ensure that there are no fingerprints or water on the bottom of the plate, and that the fluid in the wells is free of bubbles. Measure the absorbance at 450 nm immediately.
12. **Analysis**
13. **Calculations**:

Mean absorbance of the positive control should be ≥1.00

Mean absorbance of the negative control should be ≤0.10

CUT OFF value=Negative control + 0.15

1. **Interpretation of results**:

If the positive control value is ≥ 1.00 and the negative control value is ≤ 0.10, the test is valid, otherwise, the test is invalid.

If O.D. of samples < CUT OFF, the test samples are considered negative.

If O.D. of samples ≥ CUT OFF, the test samples are considered positive.

1. **Precautions**
2. Before using the kit, centrifuge the **tubes** to bring down the contents trapped in the lid
3. **Avoid foaming** or **bubbles** when **mixing** or **reconstituting** components.
4. If crystals have formed in the concentrated **Wash Buffer**, warm to room temperature and mix gently until the crystals have completely **dissolved.**
5. It is recommended measuring each controls and samples in duplicate.
6. Do not let the wells uncovered for extended periods between incubation. Once reagents are added to the wells do not let the strips dry as this can inactivate the biological material on the plate. Incubation time and temperature must be controlled.
7. Ensure plates are properly sealed or covered during incubation steps.
8. Complete removal of all solutions and buffers during wash steps is necessary for accurate measurement readings
9. Do not reuse pipette tips and tubes to avoid cross contamination.
10. Do not use expired components from different kit.
11. The TMB Substrate B is easily contaminated: protect from light and work under sterile conditions when handling the TMB substrate solution. Equilibrate at room temperature prior to use. Unreacted substrate should be colorless or very yellow in appearance. Aspirate the dosage needed with sterilized tips and do not dump the residual solution back into the vial.

# BOB (BLOCKING OF BINDING) ASSAY

ELISA-based assay to detect specific Zika antibodies (Abs) in sera or plasma of Zika virus (ZIKV)-immune individuals and to differentiate from prior exposure to DENV. The assay is based on the principal that the presence of antibodies in the serum of individuals that react with the non-structural-1 (NS1) protein of ZIKV inhibit the assay signal, which is based on recognition of ZIKV NS1 by the monoclonal antibody (ZKA35). Of note, ZKA35 specifically binds ZIKV NS1 but not dengue virus (DENV) NS1.

REAGENTS

- ZKA35-HRP labeled (Absolute antibody #Ab01036-10.0-BOB)
- Cold (non-conjugated) antibody: ZKA35-rIgG1 (unlabeled; Absolute antibody #Ab01036-10.0-CTL)
- Antigen (ZIKV NS1 e.g. Meridian #R01636 or Native Antigen Company # ZIKV-NS1-100 )
- Phosphate Buffered Saline (PBS)
- ubstrate: SureBlue Reserve TMB 1-Component Micro well Peroxi (e.g. Bioconcept #5120-0083)
- Stop solution: 1% HCl
- Nunc-Immuno plates - 96-well plate, MaxiSorp (e.g. Sigma #M9410-1CS) (=Nunc 439454)

(Alternative plate e.g. Product #9018: Corning 96 Well Clear Flat Bottom Polystyrene High Bind Microplate, 25 per Bag, without Lid, Nonsterile)

Note: Do not use 384 well plate for this assay, as the background would be too high.

| *Blocking buffer/Diluent* | 1% BSA *(e.g. Sigma, #* A9430*)* in PBS |
| --- | --- |
| *Bicarbonate buffer* | 1.59g Na2CO3 (e.g. Sigma-Aldrich #71345)  2.93g NaHCO3 (e.g. Sigma, #71627)  0*.2g NaN3 (e.g. Sigma, #71290)*  1 L milliQ-H2O  pH 9.6 |
| *Washing buffer*: | *0.5% PBS*  *0.05% Tween 20* |

**(I) DETERMINATION OF ZKA35-HRP WORKING DILUTION (EC70)**

To determine EC70 of ZKA35-HRP concentration to be used in the competition assay for binding to coated ZIKV NS1 in your experimental condition:

- Coat plates with antigen (1 μg/ml ZIKV NS1) in PBS, 50 μl/well
- Incubate over night at 4°C, plate must be sealed
- Wash plates 2x with Washing buffer (250 μl/well)
- Add blocking buffer, 200 μl/well
- Incubate 1 h at room temperature
- Wash plates 2x with Washing buffer (250 μl/well)

Add **50 μl sample/well** of: (1) sera/plasma to test (1:10 diluted in diluent), duplicates recommended

2) 3 wells **negative control**: control plasma/sera diluted 1:10 in assay diluent or diluent only

3) 3 wells **positive control:** 5 μg/ml ZKA35 mAb in control plasma/sera that was diluted 1:10 in assay diluent)

- Row 1 and 2: diluent
- Row 3 and 4: Negative control serum diluted 1:10 in diluent
- Incubate 1 h at room temperature
- Do not wash
- Add ZKA35-HRP: starting dilution 1:10 , 1:3 serial dilution of 12 points in diluent, using **50 μl ZKA35-HRP/well** (***)
- Incubate 15 min at room temperature
- Wash plates 4x with Washing buffer (250 μl/well)
- Dispense substrate solution SureBlue Reserve TMB, 40 μl/well
- Incubate at room temperature (time depending on signal: until reaching a visible bright blue color, e.g. 10-15 min. After adding the stop solution (below), a final OD value of 2-3 should be reached in the upper plateau of the dilution series.)
- Dispense stop solution 1% HCl, 40 μl/well
- Read absorbance at 450 nm
- Determine the concentration of ZKA35-HRP to be used in the BOB assay corresponding to 70% of the maximal OD level by interpolating a curve fitted with a 4-parameter nonlinear regression. Consider the

ZKA35-HRP input dilution (***), see example data, below.

Example of plate layout

- Incubate 1 h at room temperature
- DO NOT WASH
- Add ZKA35-HRP at the concentration of the amount corresponding to 70% of the maximal OD level (EC70 established above (***)), 50 μl/well (in ALL wells, including negative and positive controls)
- Incubate each plate 15 min at room temperature
- Wash plates 4x with Washing buffer (250 μl/well)
- Add substrate solution SureBlue Reserve TMB, 40 μl/well
- Incubate at room temperature
- Dispense stop solution 1% HCl, 40 μl/well
- Read absorbance at 450 nm
