# Supplementary material for: Seroprevalence of Yellow fever, Chikungunya, and Zika virus at a community level in the Gambella Region, South West Ethiopia
Source: PLoS One. 2021 Jul 8;16(7):e0253953. doi: 10.1371/journal.pone.0253953 (PMC8266044; doi:10.1371/journal.pone.0253953)
Supplement: S1 Questionnaire — (DOCX) [file pone.0253953.s002.docx]

**በትንኝ አማካኝነት ስለሚተላለፉ የቫይረስ በሽታዎች የተጋላጭነት የዳሰሳ ጥናት በጋምቤላ ብሔራዊ ክልላዊ መንግስት ፣ ደቡብ ምዕራብ ኢትዮጵያ**

**ማህበረሰብ አቀፍ የደም ናሙና ጥናት (አማርኛ ቅጂ-Amharic copy)**

የተሳታፊ ሙሉ ስም--------------መለያ-----ወረዳ------ቀበሌ--------መንደር------ቀን……/ ----/…..

***እባክዎትን ከዚህ በታች ላሉት ጠቅላላ መጠየቅ ያለዎትን መረጃ ያካፍሉን***

| 1 | ፆታ: 1. ወንድ 2. ሴት |
| --- | --- |
| 2 | እድሜ: _________________ዓመት |
| 3 | ብሔረሰብ: 1.ኑየር 2.አንዋ 3. ኮሞ 4.ኦፖ 4.ሌሎች (ይግለጹ) ________________ |
| 4 | ሐይማቦት: 1. ፕሮቴስታንት 2.ኦርቶዶክስ 3.ሙስሊም 4. ካቶሊክ 5.ሌሎች (ይግለጹ) _______ |
| 5 | የትምህርት ደረጃ: 1.ኢመደበኛ 2. መደበኛ |
| 6 | ስራ: 1.እርብቶ አደር 2.ከፊል አርብቶ አደር 3.ሌላ (ይግለጹ) ______ |
| 7 | በዚህ ቀበሌ የኖሩበት ጊዜ: ____________________________ |
| 8 | በሌሎች ሀገራት ወይም ክጋምቤላ ክልል ውጭ ኖረው ያውቃሉን ? 1. አዎ 2. በፍጹም |
| 9 | ለትያቄ ቁጥር 8 መልስዎ አዎን ከሆን የትና መቼ ለምንስ ያክል? የት: _______________ መቼ ________________ ልምን ያክል--------- |
| 10 | ወደ ደቡብ ሱዳን፣ ኬንያ፣ ዩጋንዳ እና ሌሎች የኢትዮጵያ አካባቢዎች ሄደው ያውቃሉን?. 1.አዎ 2. በፍጽም |
| 11 | አወን ከሆን መልስዎ የት. __________________;መቼ ___________________ |
| 12 | በጫካ ውስጥ ስራም ሆን በተደጋጋሚ ይጓዙ ነበርን: 1. አዎ 2.በፍጹም |
| 13 | ለጥያቄ ቁጥር 12 አወን ከሆነ የት?_________ |
| 14 | ይችን ትንኝ ያውቋታልን (የኤደስ ትንኝን በፎቶ በማሳየት): 1.አዎ 2. በፍጹም |
| 15. | አወን ከሆን ልጥያቄ ቁጥር 14 መልስዎ የት ነው የምትራባዉ ? _________________________________ |
| 16 | በዚች (ኤደስ ትንኝ በምስል በተደገፈ) ተነክሰው ያውቃሉን?: 1.አዎ 2.በፍጹም |
| 17 | ለጥያቄ ቁጥር 16 መልስዎ አወን ከሆነ የምትናደፈው መቼ ነው? ፤ 1. ማታ ቤት ውስጥ; 2. ቀን ቤት አካባቢ; 3.ቀን ጫካ ውስጥ ; 4. ሌላ ቦታ (ይጠቀስ) _______________ |
| 18 | ለጥያቄ ቁጥር 16 መልስዎ አወን ከሆነ በዚች ትንኝ መነደፍ በሽታ ያመጣልን : 1. አዎ 2. በፍጹም 3. አላውቅም |
| 19 | ለጥያቄ ቁጥር 18 መልስዎ አወን ከሆነ ምን አይነት በሽታ ?________________________ |
| 20 | ለረጅም ግዜ የተቆራኝዎት በሽታ አለን : 1. አዎ 2. በፍጹም |
| 21 | ለጥያቄ ቁጥር 20 መልስዎ አወን ከሆነ የበሽታውን ስም ቢጠቅሱልን : ________________________________ |
| 22 | ባካቢዉ እንደ ጦጣ ዝንጀሮ ይኖራሉን ? 1. አዎ 2.በፍጹም |
| 23 | ለቢጫ ወባ በሽታ ክትባት ተከትበው ያውቃሉን: 1.አዎ 2. በፍጹም |

መረጃውን የሰበሰበው ሰው ስም _____________________ ቀን ____________ ፊርማ ____________
