# Supplementary material for: Seroprevalence of Yellow fever, Chikungunya, and Zika virus at a community level in the Gambella Region, South West Ethiopia
Source: PLoS One. 2021 Jul 8;16(7):e0253953. doi: 10.1371/journal.pone.0253953 (PMC8266044; doi:10.1371/journal.pone.0253953)
Supplement: S2 Questionnaire — (DOCX) [file pone.0253953.s003.docx]

**Risk factor assessment on arboviruses exposure in Gambella Regional State, South Western Ethiopia**

**Community based serological test)**

**(English copy)**

Name of Participant: _________________code.________Woreda________Kebele____________ Village_____________________ Date _____/_____/_________

***Please kindly provide information for the following general questions***

| 1 | Sex: 1. Male 2. Female |
| --- | --- |
| 2 | Age: _________________years |
| 3 | Ethnicity: 1. Nuer 2. Anywaa 3. Komo 4. Opow 5. Others (specify) ________________ |
| 4 | Religion: 1. Protestant 2. Orthodox 3. Muslim 4. Catholic 5. Others(specify) _______ |
| 5 | Educational Status: 1. Informal 2. formal |
| 6 | Occupation: 1. Pastoralist 2. Agro pastoralist 3. Others (specify) ______ |
| 7 | Duration of stay in this kebele: ____________________________ |
| 8 | History of residence in other countries 1.Yes 2. No |
| 9 | If Q8 yes, where and when: Where _______________When________________months/years |
| 10 | Do you have any travel history to other country/area (like South Sudan, Kenya, Uganda/other areas within Ethiopia): 1. Yes 2. No |
| 11 | If yes where and when: Where __________________; When ___________________ |
| 12 | History of working/traveling to areas like forest: 1. Yes 2. No |
| 13 | If Q12 yes, when _________ |
| 14 | Do you know this mosquito (Aedes mosquitoes’ picture demonistration): 1. Yes 2. No |
| 15. | Where they (Aedes mosquito) are found/breed? _________________________________ |
| 16 | History of biting by this mosquito (*Aedes* mosquitoes’ picture observation): 1. Yes 2. No |
| 17 | If Q16 yes, where and when: 1. Night at home; 2. Day time around home; 3. Day time in Forest area; 4. Other specify _______________ |
| 18 | If Q 16 yes, do you think biting by this mosquito causes a disease: 1. Yes 2. No 3. I don’t know |
| 19 | If yes to question 18, what type of disease?________________________ |
| 20 | Any history of chronic diseases: 1. Yes 2. No |
| 21 | If Q 20 yes, mention the disease: ________________________________ |
| 22 | Are there primates (monkey and apes around your residence or grazing areas)? 1. Yes 2. No |
| 23 | History of vaccination for yellow fever (check list/card): 1. Yes 2. No |

Date collected by_____________________ date____________signature____________
